# Orthopedic applications of 3D printing in canine veterinary medicine

**DOI:** 10.3389/fvets.2025.1582720

**Published:** 2025-04-23

**Authors:** Claire Thomas, Pierre Amsellem, David Nascene, Yu-Hui Huang

**Affiliations:** ^1^Medical School, University of Minnesota, Minneapolis, MN, United States; ^2^College of Veterinary Medicine, Colorado State University, Fort Collins, CO, United States; ^3^Department of Radiology, University of Minnesota, Minneapolis, MN, United States

**Keywords:** 3D printing, orthopedics, additive manufacturing, intraoperative guide, presurgical planning, anatomic modeling, canine surgery

## Abstract

**Objective:**

This case series investigates the application of 3D printing in veterinary orthopedic surgeries, emphasizing its potential to enhance preoperative planning, intraoperative precision, and postoperative outcomes.

**Animals:**

Three canines—German Shepherd, Basset Hound, and Labrador Retriever—were included in this study.

**Materials and methods:**

Three canine cases involving complex orthopedic deformities were selected to illustrate different uses of 3D printing in veterinary surgery. CT scans were segmented using Materialise Mimics 26.0, followed by virtual surgical planning and creation of 3D printed models and guides.

**Results:**

In Case 1, a 2-year-old German Shepherd with a congenital right tibial deformity underwent successful surgical correction aided by a preoperatively prepared external fixator frame, saving approximately 1 h of OR time. In Case 2, a 1-year-old Basset Hound with a left antebrachial deformity had a double wedge osteotomy performed with the assistance of patient-specific cutting and reconstruction guides, leading to optimal alignment and reduced surgical time. Case 3 involved a young, less than 1-year-old Labrador Retriever rescue with severe bilateral tibiofemoral deformity, where 3D printed models helped the surgeon determine that surgery was not the best option, potentially preventing a poor outcome.

**Clinical relevance:**

This case series highlights the transformative potential of 3D printing in veterinary orthopedic surgery, illustrating its ability to improve aid surgical outcomes, reduce operative times, and be a valuable tool in preoperative decision-making. This technology allows for tailored surgical interventions, enhancing the precision and effectiveness of treatment plans in veterinary medicine.

## Introduction

Additive manufacturing, commonly referred to as 3D printing, is the process by which virtual digital files can be manifested as physical objects. Within medicine, advanced volumetric imaging, such as computed tomography (CT) and magnetic resonance imaging (MRI), can be virtually translated from 2D sliced images to reconstruct 3D objects. These 3D objects can then be 3D printed in materials of varying qualities using additive manufacturing technologies.

Commonly printed models include physical representations of complex anatomy utilized for presurgical planning (1–4), anatomical education (5–9), and simulation (9–13). With the development of 3D printing materials that can be sterilized, more intraoperative models are being produced and utilized such as cutting and reconstruction guides to aid precise osteotomies and hardware placement (5, 9, 11). These techniques are already heavily adopted for human medical care, particularly in complex or unique cases, but there is still limited literature supporting their use in veterinary medicine. Veterinary medicine stands to benefit greatly from 3D printing through enhanced understanding of animal pathology for veterinary surgeons, trainees, and animal owners.

Historically, the anatomical heterogeneity across diverse animal species has posed challenges in the field of veterinary orthopedics. The advent of medical 3D printing has provided means to navigate these challenges by enabling the translation of high-fidelity medical imaging datasets into tangible 3D anatomic models. Advancements in veterinary medicine have witnessed the beginning stages of integration of 3D printing technologies, catalyzing transformative changes in the approach to a limited number of complex orthopedic cases (10–13).

A systematic review of veterinary orthopedic literature by Memarian et al. found benefits of 3D printing technologies in education, preoperative planning, client communication, custom-made orthopedic implants (total joint replacement—hip, knee, patellar groove, and elbow), limb-sparing surgery, corrective osteotomies, arthrodesis, as well as customized scaffolds for forays into 3D bioprinting (11). Kamishina et al. demonstrated clinical benefits of using patient-specific titanium plates in 83.3% (15/18) of spinal stabilization surgeries with most benefit being gained from accurate placement of screws and minimization of complications during technically demanding parts of surgery (12). Fracka et al. found that patient-specific guides had the potential to improve the accuracy of tibial and femoral cut alignment in canine total knee replacement (TKR), especially in cases where surgeons had limited prior experience with TKR surgery (13). Altwal et al. highlighted presurgical modeling as a major emerging use of 3D printing in small-animal surgery, supporting the need for case-based evidence such as we will present here (1).

This case series will examine three distinct applications of 3D printing in veterinary orthopedics. We emphasize the creation of detailed anatomic models and their role in guiding orthopedic surgeries with heightened precision. We offer an example of the diagnostic potential of 3D printing as a preoperative decision-making tool, assessing its capacity to potentially prevent unnecessary surgeries and enhance overall patient care.

## Materials and methods

### Case selection

Three canines—German Shepherd, Basset Hound, and Labrador Retriever—were included in this study. These cases were selected from our recent clinical caseload to highlight a range of applications where 3D printing was utilized. Owner consent was obtained for all three canines in this case series.

### Imaging and segmentation

Volumetric CT images were obtained at a slice thickness of 1 mm or thinner to optimize accuracy. To begin, the Digital Imaging and Communications in Medicine (DICOM) data from these scans was anonymized and uploaded into Materialise Mimics 26.0 segmentation software (Leuven, Belgium) or DICOM to PRINT® (D2P) (3D Systems, Inc. Rock Hill, SC), both of which are FDA-cleared—a necessity given that 3D anatomical models are a class II medical device and require regulatory clearance. Once in the software, specific anatomy was isolated by selecting Hounsfield unit (HU, a measure of radiodensity) ranges. Soft tissues tended to be in the range of 40–50 HU, bone >1,000 HU, and fat −100 to −155 HU. Each CT slice was manually visualized to ensure that anatomic integrity and accuracy were maintained and serve the purpose desired for the eventual 3D model. Once the segmentation was complete, stereolithography (.STL) files were exported and overlaid on the original scan to verify the accuracy of the contours against the imaging data.

### Computer aided design (CAD)

When necessary, the. STL file was exported to Materialise 3-matic 13.0 (Leuven, Belgium) CAD software program to manipulate the files such as creating magnet holes for part retention, smoothing or hollowing the models, adding support structures to allow for accurate printing, and creating surgical guides that are shaped to the specific anatomy in an individual case. For these design tasks, we created parts using primitive shapes that were then morphed to the contours of the anatomy they were correcting. Boolean operations of addition and subtraction, which allow separate parts to become a union or to ensure there is no overlap, were heavily utilized to perform these functions. In cases where magnet holes were necessary, a tolerance of ± 0.2% of the hole size was incorporated to account for inter-print variability in accuracy.

### Surgical planning

These 3D models were uploaded to Materialise Viewer (Leuven, Belgium), an online platform used to view and mark. STL files, and shared with the veterinary surgeons for surgical planning. In this software, the veterinarian could mark which planes and angles were desired for cutting, which pieces of anatomy they would theoretically want to manipulate during surgery, and, most importantly, it served as an easy point of collaboration that could be easily referenced across geographies.

### 3D printing

Once anatomy and/or cutting guides were finalized and agreed upon, .STL files were uploaded to Formlabs 3B printer’s proprietary software PreForm v3.21.0 (Formlabs Inc. Somerville, MA). Within this software, support structures were automatically added and ideal printing orientations were algorithmically chosen by the software to optimize print quality and shorter printing times. All 3D models were printed using a Formlabs Form 3B desktop 3D printer (Formlabs Inc. Somerville, MA), which utilizes stereolithography (SLA) printing with resin. These SLA resin 3D printers used a laser to cure liquid resin into hardened plastic in a process called photopolymerization. Osseous anatomic models were printed using Formlabs White resin and intraoperative guides were printed using Formlabs Biocompatible Surgical Guide resin. Models and guides were provided to the veterinarian surgeons 1–2 days prior to surgery to allow for adequate preparation.

## Results

### Case 1

A 2-year-old German Shepherd with congenital right tibial deformity was undergoing surgical correction and the orthopedic veterinarian consulted for an anatomic model. Segmentation isolated the right tibia and fibula and took approximately 0.5 h to segment, 13 h to print, and used 32 mL of white resin.

**Figure 1:**
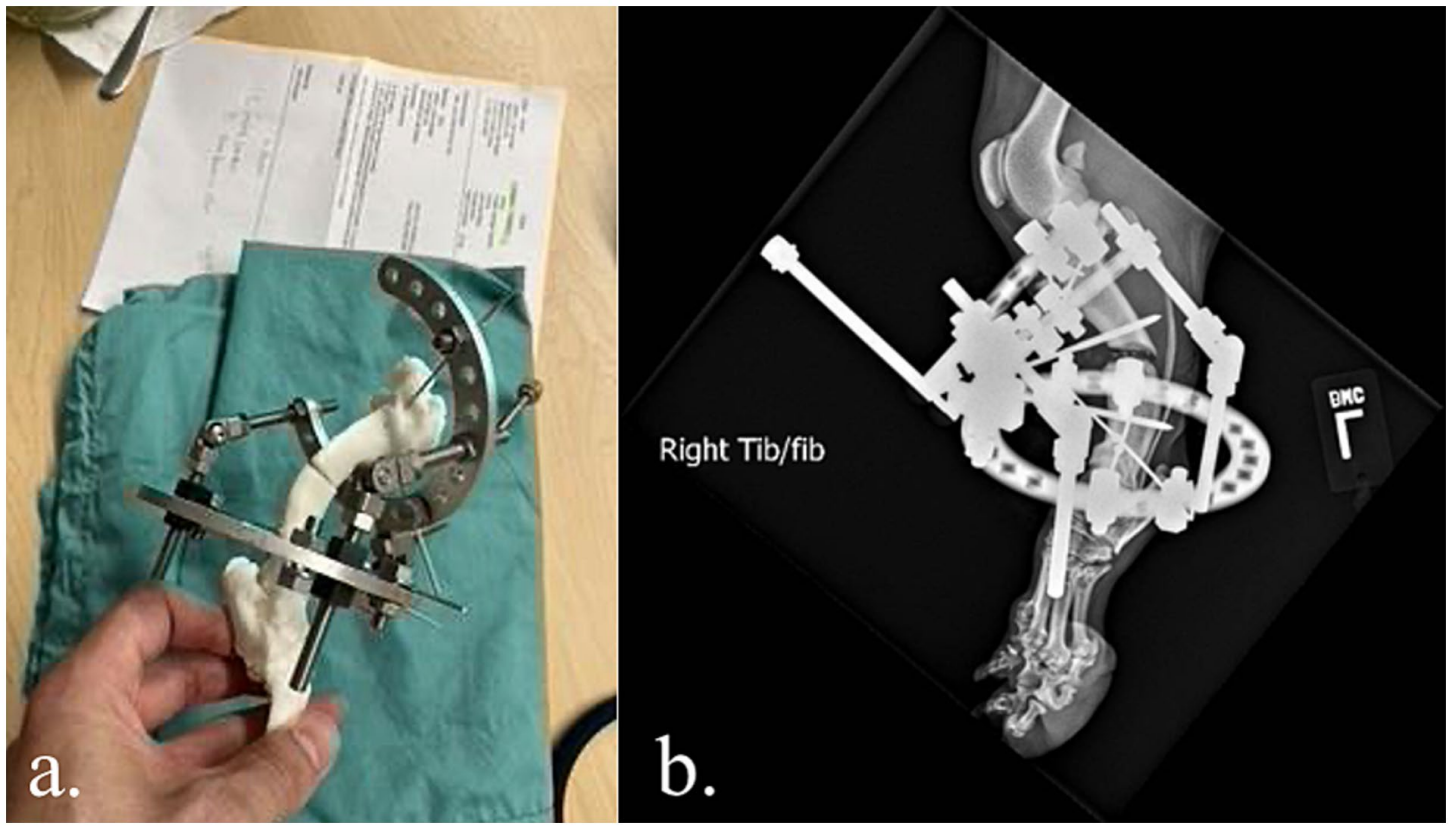
**(a,b)** Complex external fixator construct applied to a 3D printed model prior surgery. Satisfactory alignment following external fixation of a congenital right tibial deformity in a 2-year-old German Shepherd.

The veterinarian used the anatomic model for surgical planning and they were able to prepare the complex external frame (Imex external fixator, Longview, TX) ahead of time (Figure 1a) and achieve satisfactory alignment (Figure 1b). This saved approximately 1 h of OR time compared to the veterinarian’s historic average for similar cases. This canine is doing well 12 months after surgery despite the lack of bony union and bone plating after external fixator removal.

### Case 2

**Figure 2:**
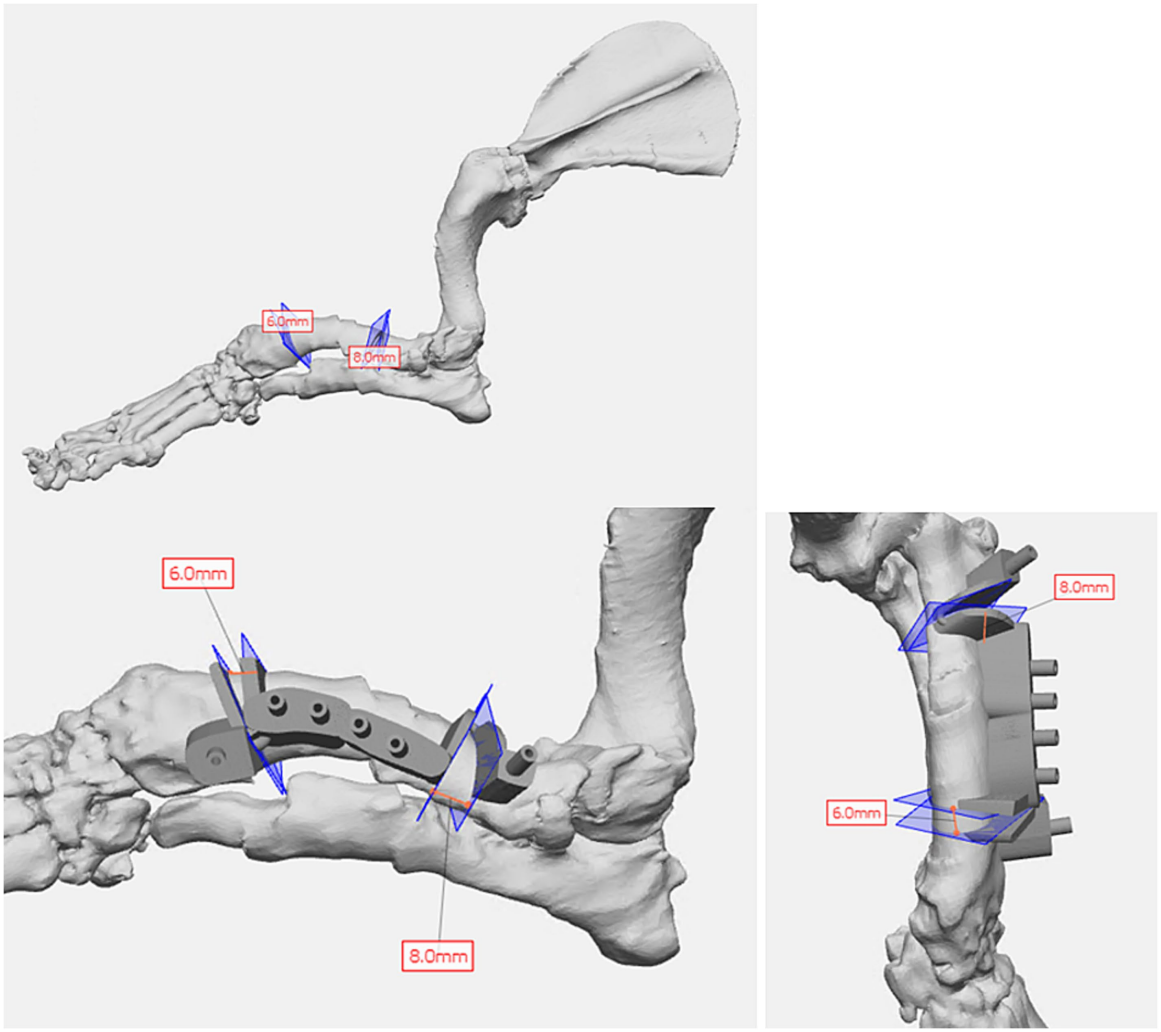
Virtual surgical planning of double wedge osteotomies and cutting guide design in a 1-year-old Basset Hound with left antebrachial deformity requiring surgical correction of curvature and rotation.

A 1-year-old Basset Hound with left antebrachial deformity was undergoing surgical correction. The orthopedic veterinarian requested virtual surgical planning, anatomic models, and surgical guides for intraoperative pin placement to correctly approximate bone segments. Initially, the forelimbs were segmented, which took approximately 0.5 h. Virtual surgical planning was performed with the veterinarian surgeon for double wedge osteotomies with desired locations, orientations, and width of the wedges. The bone segments were then aligned with the feedback of the veterinarian surgeon to optimally correct for the curvature and rotation (Figure 2).

**Figure 3:**
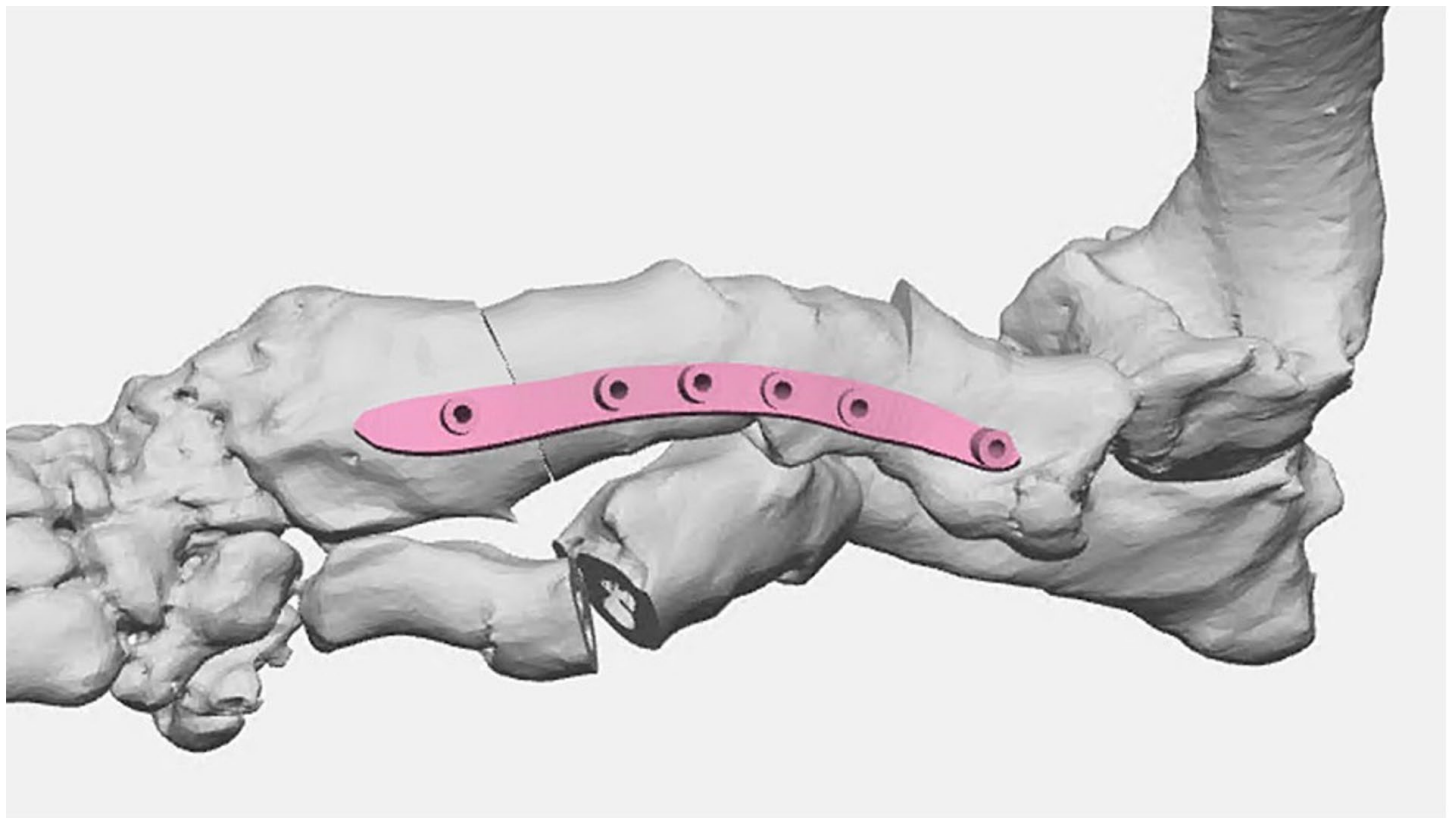
Surgical planning of bone segment positions post virtual osteotomies with reconstruction guide design for optimal surgical correction.

**Figure 4:**
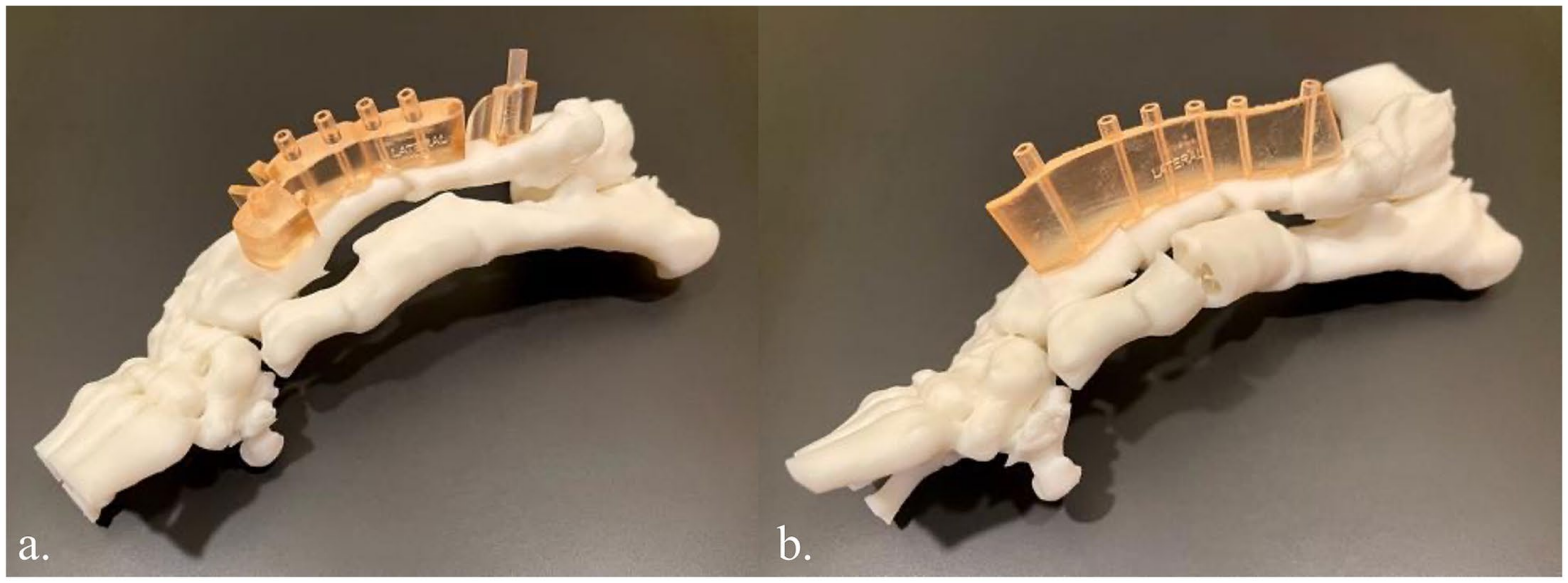
**(a)** 3D printed innate left antebrachial deformity in white resin and the cutting guide in biocompatible surgical guide resin (light orange). **(b)** 3D printed left antebrachial reconstruction per the virtual surgical plan in white resin and the reconstruction guide in biocompatible surgical guide resin.

**Figure 5:**
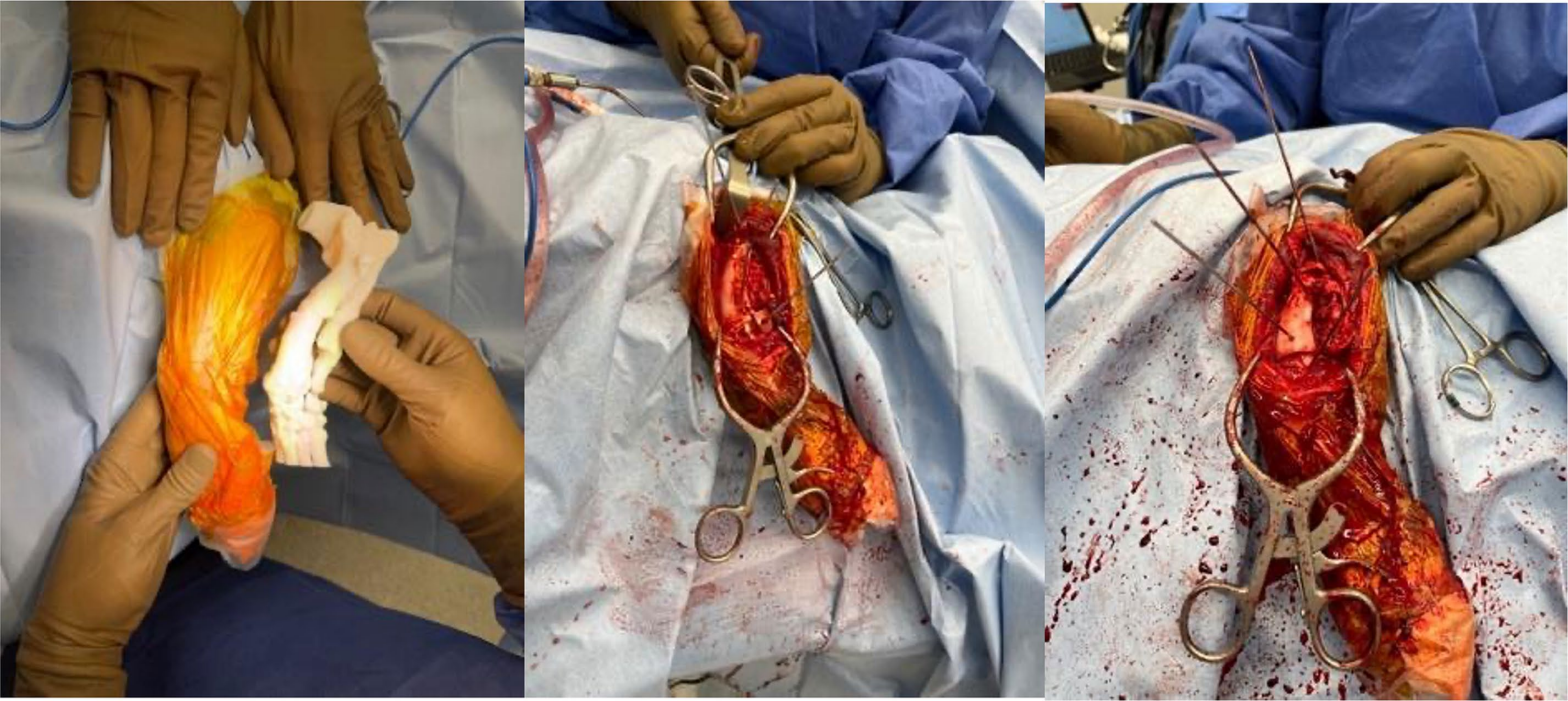
Intraoperative photos of results of utilizing sterilized anatomic model, cutting guide and reconstruction guide for left antebrachial surgical correction.

**Figure 6:**
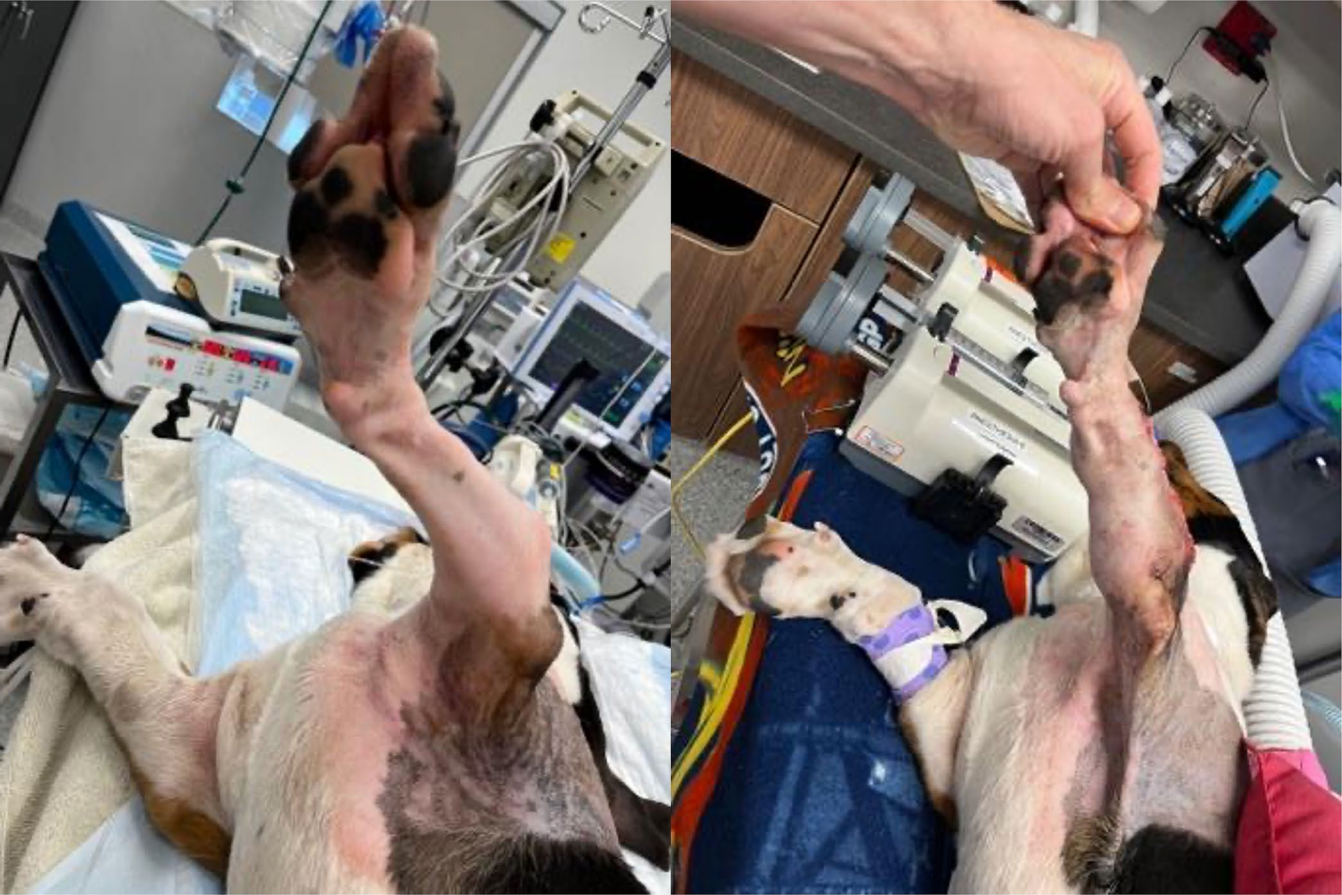
Before and after photos showing reduced antebrachial curvature and rotation of the left forelimb.

Once the plan was finalized, a single piece cutting guide was designed for the planned double wedge osteotomy with pin holders according to the anticipated tissue exposure as well as the need for the fixation pins to be placed laterally (Figure 2). A reconstruction guide was designed to align the bone segments in the planned positions using the same fixation pins (Figure 3). The cutting guide and reconstructed model with the reconstruction guide were printed in biocompatible surgical guide FormLabs resin (Figures 4a,b). The reconstructed anatomy took 11 h to print and used 88 mL of white resin. The cutting guide took 5.5 h to print and used 49 mL of biocompatible surgical resin. These models were printed concurrently on separate printers. The models and guides were sterilized for intraoperative use with the placement of the cutting guide to execute the double wedge osteotomies. After the osteotomies, the reconstruction guide was applied to align the bone segments as planned for plate fixation (Figure 5). Proper alignment was achieved and the severe curvature of the left forelimb was corrected (Figure 6).

The surgeon reported that the models and guides saved him over 1 h of surgery time and ensured optimal surgical correction and outcome. He also remarked how he was able to use anatomic landmarks on the model to guide him during surgery where landmarks became obscured by tissue and blood. This canine is doing well over 12 months after surgery.

### Case 3

**Figure 7:**
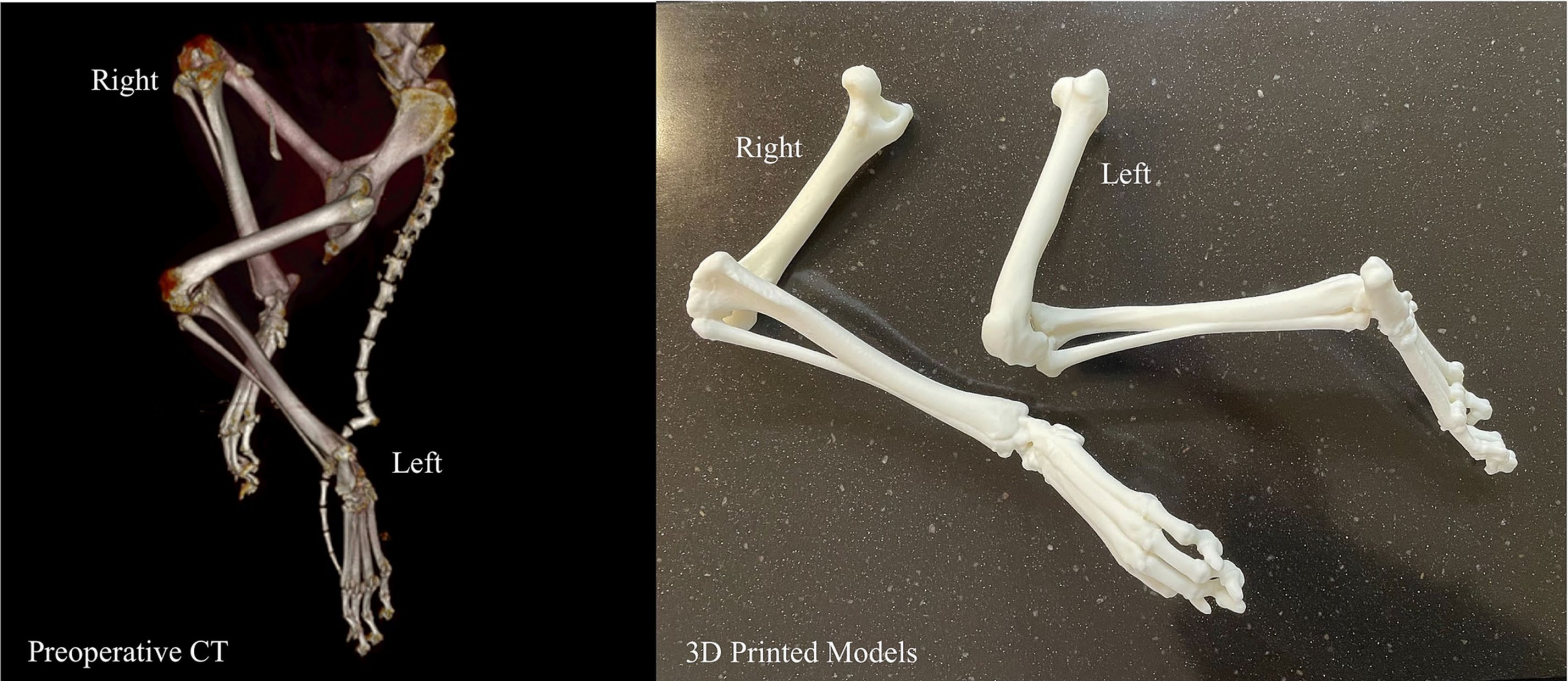
Volume rendering and 3D printed models of bilateral tibiofemoral malalignment for surgical planning.

A young, less than 1-year-old, Labrador Retriever rescue with severe bilateral hindlimb deformity was being evaluated for possible surgical correction and an anatomic model was requested. The volume rendering of his hindlimbs from his CT demonstrated severe malalignment of his tibiofemoral joints bilaterally (Figure 7). The hindlimbs were segmented and modified for magnetic retention to maintain the native anatomic orientation. The 3D model assisted the surgeon in determining that surgical correction was not the optimal treatment plan and could possibly result in a worse outcome.

A summary of all canines included in this study can be found in Table 1.

**Table 1:** A summary table of all three canines included in this case series that includes age, breed, diagnosis, 3D model purpose, and outcome of case after using the 3D model.

Case	Age	Breed	Diagnosis	3D Model purpose	Outcome
Case 1	2 years	German Shepherd	Right tibial deformity	Presurgical planning and preoperative fitting of external frame	Satisfactory surgical alignment and decreased time spent in surgery
Case 2	1 year	Basset Hound	Left antebrachial deformity	Presurgical planning and intraoperative guide	Satisfactory surgical alignment and decreased time spent in surgery
Case 3	<1 year	Labrador Retriever	Bilateral hindlimb deformity	Presurgical decision making	Surgery cancelled

## Discussion

This case series of three orthopedic applications of 3D printing in veterinary medicine highlights the potential benefits of utilizing this technology, as well as providing specific examples of instances where it can effectively be used. Specifically, 3D printing in veterinary medicine can be used to preoperatively prepare external frames for complex orthopedic injuries, create detailed anatomic models for osteotomies as well as surgical guides to steer pin placement for adequate realignment, and provide an avenue whereby operative judgment can be exercised ahead of time, possibly preventing unnecessary surgery. These models assisted veterinary surgeons in optimizing their surgeries through enhanced anatomic understanding, better preparation, and confidence in their treatment plans.

Notably, despite optimal alignment and reduced surgery time, Case 1 resulted in a non-union at the osteotomy site (treated with an external fixator without subsequent plating). The canine remained clinically stable and pain-free at 12 months, indicating a functional outcome even without complete bony union. Similar to our results in Case 2, a previous study by Jeong et al. planned a corrective osteotomy for treatment of a medial patellar luxation in a canine using a 3D-printed bone model for pre-contouring and demonstrated improvement of limb function without relaxation of the patella through increased surgical accuracy (10). In Case 3, the anatomic models aided in decision-making to avoid a likely poor surgical outcome. Our cases add to the existing literature that supports the notion that 3D printed models can reduce OR time, decrease time under anesthesia, and avoid potential complications from improvising during surgery.

The veterinary surgeons in our unique cases remarked on how the printed physical models allowed them to examine the anatomy from different angles in order to create a treatment plan that was optimized for each specific canine. Though this case series is small, in these specific instances, the 3D models decreased operation time and, in some cases, allowed the veterinary surgeons to have a tactile appreciation during the surgery. Surgery requires an appreciation for haptic feedback, which allows opportunity for the insertion of 3D printed models into the presurgical and intraoperative process. Previous 3D printing case series have consistently supported the use of anatomic models in determining the margins of a mass, both before and during surgery, which assists with successful resection with proper margins (1, 2, 11). This can be especially helpful in veterinary medicine where numerous species of animals, as well as breeds within those species with varying anatomic composition, exist. With this variety, anatomic landmarks during surgeries may largely differ, and can require intimate understanding of spatiality.

An important consideration in orthopedic-oriented medical 3D printing is that imaging parameters are commonly limited by slice thickness. Currently, most of the diagnostic imaging parameters are not optimized for 3D printing. The thicker the slices, the less detailed the models. Artifacts, especially scatter artifacts related to existing metallic hardware, can be a barrier in orthopedic care where they are more commonplace. Even though metal artifact reduction algorithms can improve image quality, it can also sometimes generate new artifacts. Thus, it is important to review the images with a radiologist or imaging specialist in multiplanar reformats to avoid misinterpreting newly generated artifacts as true anatomy or pathology. Additionally, the specific need for the model determines which imaging modalities and parameters are most appropriate. An imaging specialist, such as a radiologist, is uniquely qualified to assess parameter appropriateness.

Our study was limited by being a three-case series, which makes these results difficult to extrapolate. As a case series without a control group, we cannot definitively quantify the advantages. Rather, our report is intended only to demonstrate the feasibility and potential benefits of this technology. The outcomes observed, while promising, should be interpreted as illustrative examples that will require validation in larger studies. Current general limitations to 3D printing in veterinary surgery include cost and necessary printing time and post-processing. Segmentation itself is completed using software that can range in cost from free to thousands of dollars per year for one license. When used for medical purposes, it is encouraged to utilize software that is specialized for use with advanced imaging for the purpose of optimizing image data and achieving accuracy.

Furthermore, desktop 3D printers are becoming increasingly accessible and affordable. These desktop 3D printers are recommended for simple anatomic models mainly due to these printers typically being single-nozzle with a few color options available and less capability overall. Industrial grade 3D printers cost a minimum of a few thousand dollars and range up to hundreds of thousands of dollars. These printers tend to have more material options, color varieties, and accurate printing specifications, often down to microns. However, the more accuracy a model requires, the thinner the 3D printed layers will be, which will increase the amount of time needed for printing algebraically. Once printed, these models at a minimum usually require separation of the 3D object from its 3D printed support structures, as well as the possibility of additional sanding, painting, magnet insertion, etc. These limitations are being addressed by newer 3D printers, but still remain a very present constraint for accessibility and usage.

Future applications of 3D printing in orthopedic veterinary surgery cases should quantify data by assessing data such as blood loss, OR time, time under anesthesia, and complication rate against a matched control group of canines where 3D printed models are not utilized. Additional information can be obtained from studies specifically studying anatomic models and usage in presurgical planning or surgical cutting guides and their possible optimization of the surgical process. These two usage scenarios often go hand in hand. Implementation of this technology in veterinary medicine should seek to develop case series with larger study populations so that both internal and external validity can be assessed. Furthermore, a wide variety of usages in diverse animal populations can be investigated to continue finding productive applications of additive manufacturing. Supplemental research can also aim to explore other burgeoning technologies that are related, such as virtual reality, augmented reality, and photogrammetry.

In conclusion, the integration of 3D printing technology into veterinary orthopedic surgeries has the potential to reshape traditional treatment paradigms and can possibly elevate the standard of care for animal patients. The multifaceted applications explored in this paper underscore the substantial impact 3D printing can have on presurgical planning, intraoperative precision, and postoperative assessment. The customization of external frames, surgical guides, and implants through 3D printing stands out as a hallmark advancement, allowing veterinary surgeons to tailor interventions to the unique anatomical nuances of each patient. This level of personalization not only elevates the precision of surgeries, but also contributes to postoperative recoveries that align more closely with the individual needs of the animal.

## Data Availability

The raw data supporting the conclusions of this article will be made available by the authors, without undue reservation.
